# Identification of Molecular Markers Associated with Verticillium Wilt Resistance in Alfalfa (*Medicago Sativa* L.) Using High-Resolution Melting

**DOI:** 10.1371/journal.pone.0115953

**Published:** 2014-12-23

**Authors:** Tiejun Zhang, Long-Xi Yu, Per McCord, David Miller, Suresh Bhamidimarri, David Johnson, Maria J. Monteros, Julie Ho, Peter Reisen, Deborah A. Samac

**Affiliations:** 1 United States Department of Agriculture, Agricultural Research Service, Plant Germplasm Introduction and Testing Research, 24106 N Bunn Road, Prosser, Washington, United States of America; 2 DuPont Pioneer, W8131 State HWY 60, Arlington, Wisconsin, United States of America; 3 Alforex Seeds, N4505 CTH M, West Salem, Wisconsin, United States of America; 4 Forage Improvement Division, Samuel Roberts Noble Foundation, 2510 Sam Noble Parkway, Ardmore, Oklahoma, United States of America; 5 Forage Genetics International, Inc. 7661 Becker Road, Davis, California, United States of America; 6 United States Department of Agriculture, Agricultural Research Service, 495 Borlaug Hall, 1991 Upper Buford Circle, Saint Paul, Minnesota, United States of America; National Key Laboratory of Crop Genetic Improvement, China

## Abstract

Verticillium wilt, caused by the soilborne fungus, *Verticillium alfalfae*, is one of the most serious diseases of alfalfa (*Medicago sativa* L.) worldwide. To identify loci associated with resistance to Verticillium wilt, a bulk segregant analysis was conducted in susceptible or resistant pools constructed from 13 synthetic alfalfa populations, followed by association mapping in two F1 populations consisted of 352 individuals. Simple sequence repeat (SSR) and single nucleotide polymorphism (SNP) markers were used for genotyping. Phenotyping was done by manual inoculation of the pathogen to replicated cloned plants of each individual and disease severity was scored using a standard scale. Marker-trait association was analyzed by TASSEL. Seventeen SNP markers significantly associated with Verticillium wilt resistance were identified and they were located on chromosomes 1, 2, 4, 7 and 8. SNP markers identified on chromosomes 2, 4 and 7 co-locate with regions of Verticillium wilt resistance loci reported in *M. truncatula*. Additional markers identified on chromosomes 1 and 8 located the regions where no Verticillium resistance locus has been reported. This study highlights the value of SNP genotyping by high resolution melting to identify the disease resistance loci in tetraploid alfalfa. With further validation, the markers identified in this study could be used for improving resistance to Verticillium wilt in alfalfa breeding programs.

## Introduction

Alfalfa (*Medicago sativa* L.) is the most widely grown forage legume due to its high forage quality, broad adaptability, and its high value as a feed in dairy and beef production. Although demands for alfalfa are increasing, its production is challenged by endemic and emerging diseases. Verticillium wilt of alfalfa (VW), caused by the soilborne fungus *Verticillium alfalfae* [1] (previously *V. albo-atrum*) is a devastating disease in alfalfa and causes forage yield reductions of up to 50% in the northern United States and Canada [2]. Crop rotation may not be an effective control method because the pathogen can survive in weed species and can be spread from adjacent fields in infected plant material and on farm equipment and by wind, irrigation water, through pollen, and by insects [3], [4]. Furthermore, the fungus can be seed transmitted resulting in introduction of the pathogen into uninfested areas [5]. The best method for controlling the disease is to use resistant cultivars. A previous investigation demonstrated that the yearly benefit of using highly resistant varieties ranged from $21/ha to $44/ha [6].

The current alfalfa standard test for Verticillium wilt resistance evaluation is based on the visual observation of disease symptoms and is used in recurrent phenotypic selection in alfalfa breeding programs [7]. Because the disease affects forage yield and persistence, phenotypic selection requires multiple harvests per year and evaluation during multiple years. Thus, development of diagnostic markers associated with Verticillium wilt resistance in alfalfa would be helpful for improving accuracy and efficiency for identifying sources of resistance and accelerating breeding programs aimed at increasing disease resistance.

The primary challenge to develop molecular markers for marker-assisted selection (MAS) is the identification of markers in the target gene or tightly linked to the resistance loci and cost-effectively screening individuals in the breeding pipeline [8]. Plant resistance to diseases can be based on major, race-specific, host-pathogen recognition genes (R-genes) or multiple additive minor genes [9]. Sources of resistance based on multiple additive genes, often termed quantitative resistance, may be more durable than resistance based on single R-genes [10]. In alfalfa, quantitative trait loci (QTL) associated with resistance to Stagonospora leaf spot and root rot (*Stagonospora meliloti*) [11], anthracnose (*Colletotrichum trifolii*) [12], [13] and other traits have been reported [14], [15]. Viands [16] compared the inheritance of Verticillium wilt resistance in alfalfa plants selected from European cultivars and suggested that a major dominant gene controls resistance in cultivar Maris Kabul while more complex additive effects exist in cultivar Vertus. Larsen et al. [17] developed a real-time PCR procedure to quantify the amount of *V. alfalfae* in plant materials and found significant quantitative differences in the pathogen ‘load’ between Verticillium wilt resistant and susceptible cultivars. However, no gene or QTL has been reported for Verticillium wilt resistance in alfalfa.

Single nucleotide polymorphisms (SNPs) represent the most abundant source of variation in genomes, and are often functionally important [18], [19]. SNP-derived markers have been used as valuable tools for the genetic analysis of complex traits [20]. Recent advances in next generation sequencing and bioinformatics lead the identification of a number of SNP markers in alfalfa and the development of reliable assays to determine allelic dosage in tetraploid species [19], [21], [22]. High-throughput genotyping platforms provide valuable tools in mapping complex traits [23]–[25].

Alfalfa is an allogamous, autotetraploid species, plants are highly heterozygous with a strong inbreeding depression. Selfing gives rise to either self-sterility or lethal allelic combinations. Homozygous lines cannot be obtained and F2 populations may suffer from a genetic bias induced by the death of some genotypes [26]. Alfalfa populations maintain a greater genetic diversity compared to the related diploid species, such as the model legume, *M. truncatula*, thus requiring strategies that enable the detection of allelic dosage [20]. High-resolution melting (HRM) is a rapid and highly sensitive method for analyzing genetic variation [27]–[29]. Unlike other genotyping platforms that rely on the presence or absence of an allele, HRM classifies the marker into the polyploidy genotypic classes corresponding to allele dosages. It also offers opportunities for genotyping species with limited genomic resources because previous knowledge of the SNP variation present in a genotype is not required [20]. HRM has been used for genotyping and mapping traits in alfalfa [20] and several other tetraploid species including white lupin (*Lupinus albus* L.) [30], perennial ryegrass (*Lolium perenne* L.) [31] and potato (*Solanum tuberosum*) [32]. In this study, we performed HRM analysis for SNP genotyping alfalfa populations segregating for Verticillium wilt resistance to identify loci associated with VW resistance.

## Methods and Materials

### Plant materials and phenotyping

Two alfalfa mapping populations were developed from crosses between parents that were either resistant (R) or susceptible (S) to Verticillium wilt. A cross between the parental plants of 55V50-58(R) x 55V50-118(S) developed at DuPont Pioneer (PIO) resulted in a population of 188 F_1_ progeny. A second population from a cross between CW065006-3-13-2 (R) x CW065006-4-01-1(S) at Alforex Seeds (AFX) consisted of 164 F_1_ progeny. The four parents and F_1_ progeny were propagated by stem cuttings and three clones from each individual were evaluated for resistance to Verticillium wilt in a greenhouse assay as described below. The cloned plants were removed from propagation flats, and transplanted into a pot containing commercial potting soil (Pro-Mix). The stems were clipped to a length of approximately 5 cm once plants began to flower to generate clones of approximately the same size. The cloned populations were placed in a randomized complete block design with three replications and with each replication consisting of one plant.

Clones from both populations were inoculated with conidia from *V. alfalfae* isolates (N-29, Nyvall and 34, provided by DuPont Pioneer) mixed with equal amount and inoculated to the plants according to the standard protocol [7]. After incubation, Plants were evaluated for severity of foliar symptoms based on the standard protocol [33]. Disease severity was rated on a 1 to 5 scale with 1 =  resistant plant with no or minimal chlorosis of lower leaves; 2 =  moderate resistance with chlorosis of lower and middle leaves but no chlorosis of terminal leaves; 3 =  susceptible plant with well developed symptoms of chlorosis with necrotic and twisted terminal leaflets on at least one, but not all main stems; 4 =  susceptible plant with severe symptoms of chlorosis, necrosis, and twisting of all leaflets on all main stems; and 5 =  dead plant. The scores of three replications from the same individual were averaged to obtain mean disease severity score for each individual.

Phenotypic data were firstly analyzed using Levene's and T-tests to evaluate the equality of variance and means. Based on the assumption of equal variance, a test for normality was performed on the obtained disease severity scores of the PIO and AFX populations with the Kolmogorov-Smirnov tests using the SPSS software (http://www.ibm.com/software/analytics/spss/).

### Bulk segregant analysis

A bulk segregant analysis (BSA) approach [34] was used to identify chromosomes likely to contain markers associated with resistance to Verticillium wilt. Alfalfa leaf tissue from individuals of several populations contrasting for VW resistance, evaluated for response to *V. alfalfae* as described above, was obtained from three seed companies (DuPont Pioneer, Alforex Seeds and Forage Genetics International). DNA was extracted from young leaves of individual plants using the Fast DNA SPIN Kit (MP Biomedicals, Solon, OH) according to the manufacture's protocol. Bulks of DNA from susceptible or resistant plants were constructed from 13 synthetic populations from Alforex Seeds, two populations from Forage Genetics, and three populations from Pioneer Hi-bred. Each bulk consisted of DNA from 10 (Alforex Seeds) to 20 (Forage Genetics and DuPont Pioneer) individuals. These bulks were genotyped using 98 simple sequence repeat (SSR) markers spanning all eight alfalfa chromosomes (S1 Table). The bucks from Alforex populations were genotyped using an ABI 3730 capillary sequencer (the Samuel Roberts Noble Foundation facilities in Ardmore, OK). The Forage Genetics and Pioneer populations were genotyped using a LiCor 4300 DNA analyzer (USDA-ARS laboratory, Prosser, WA). For all SSR markers, a modified tailed primer PCR protocol was used [35], [36]. For data analysis, Chi-square values were calculated by first determining the frequency of an SSR allele across both resistant and susceptible bulks within a population. Secondly, this frequency was multiplied by the number of individuals within a bulk to obtain the expected number of individuals with the allele. Finally, the chi-square value was determined by the sum of squared differences between observed and expected values, divided by the expected value.

### DNA isolation and HRM analysis of F1 populations

Young alfalfa leaves harvested from each individual of F_1_ progeny and the parents were used for DNA extraction using the DNeasy 96 plant kit (Qiagen, Valencia, CA) according to the manufacturer's protocol. DNA was quantified with the NanoDrop ND1000 spectrophotometer (NanoDrop Technologies, Inc. Wilmington, DE), and the DNA concentration of each sample was adjusted to 0.1 µg/µl prior to PCR. A set of 172 SNP markers (S2 Table) were used for genotyping two F1 populations. The SNP primers used to screen for polymorphism between the two parents were located on chromosomes previously identified to have putative Verticillium wilt resistance loci (Table 1, chromosomes 1, 2, 4, 7 and 8) from the initial SSR screen. The primers were designed from alfalfa transcriptome sequences [19]. For high resolution melting, the standard HRM protocol was used as described in [20]. The criteria for PCR primer design includes a predicted annealing temperature (Tm) of 58–61°C, limited self-complementarity and poly-X, and PCR amplicon lengths of 100–170 base pairs (bp). Polymorphisms on two resistant and susceptible parents 55V50-58(R) and 55V50-118(S) (the PIO population) and CW065006-3-13-2 (R) X CW065006-4-01-1(S) (the AFX population) using HRM were evaluated as described below. Polymorphic markers were then used for genotyping individuals from the two F_1_ populations segregating for VW resistance. PCR reactions were performed in 96-well plates using S1000 Thermal Cycler (BIO-RAD, Hercules, CA) in a total volume of 10 µl per sample. The PCR reaction mixture consisted of 20 ng of genomic DNA, 800 pM dNTPs, 2.5 pM of forward and reverse SNP primers, 40 pM SYTO 82 orange fluorescent nucleic acid stains dissolved in DMSO (Life Technologies Carlsbad, CA), 1.0 U Taq DNA polymerase and 1 µl 10× QPCR buffer consisted of 100 mM Tris-Cl, pH8.7, 200 mM KCl, 100 mM (NH_4_)_2_SO_4_ and 25 mM MgCl_2_. The PCR program used was as follows: an initial denaturation step of 2.5 min at 94°C, followed by 45 cycles of 94°C for 5 s, 62°C for 15 s and 72°C for 10 s, with a final elongation step of 72°C for 5 minutes. Samples were then transferred to the LightCycler 480 (Roche Life Science, Indianapolis, IN) and a melting cycle was performed by increasing the temperature by 0.1°C s^−1^ from 56 to 95°C. Melting data were analyzed using the Gene Scanning Software (Roche Life Science, Indianapolis, IN). The samples with low fluorescence signals that lack a prominent melting curve were defined as negative and excluded from further analysis. They were then normalized by setting the target melting temperature ranges of 71°C to 80.5°C and 80.5°C to 88°C respectively, for the pre- and post-melting temperature. After shifting the temperature axis of the normalized melting curves at the point where the entire DNA is completely denatured (known as a temperature shift), the samples were assigned to different genotyping groups using hierarchical clustering based on the profile of their normalized melting curves and negative first-derivative melting peaks. The sensitivity level was adjusted to discriminate all visually distinguishable groups.

**Table 1:** Significant SSR markers identified via bulk segregant analysis in alfalfa populations.

Bulk/Population	Marker	Chromosome	Chi-square
CW20108^1^	MtBC01G06F3	1	5.0*
CW065006^1^	MtBC01G06F3	1	4.0*
V9^2^	MTBB30E02	1	4.0*
V9^2^	BE89	2	4.0*
V9^2^	Mt1D06	2	4.0*
V9^2^	BG28	2	4.0*
V9^2^	AW261	4	6.67**
V11^2^	AW261	4	6.0*
CW065006^1^	AL108	7	4.05*
CW20108^1^	AW177	7	4.0*
CW20108^1^	AW212	7	4.17*
CW058071^1^	AW212	7	4.0*
CW065006^1^	BE74	7	5.25*
CW29098^1^	BF141	7	6.75**
CW065006^1^	BF141	7	4.05*
CW064004^1^	BF56	7	4.0*
PioneerV2^3^	BG288	7	5.87*
CW064004^1^	BG288	7	4.0*
CW060097^1^	AW255	8	6.75**
Oneida VR^1^	AW255	8	5.8*

Note: ^1^Alforex population. ^2^Forage Genetics International population. ^3^DuPont Pioneer population. *Significant at p<0.05 with 1 degree of freedom. **Significant at p<0.01 with 1 degree of freedom.

### Principal component analysis

Principal component analysis (PCA) of the SNP data from the F_1_ individuals was performed using SAS PROC PRINCOMP (SAS Institute Inc., Cary, NC). To display results, principal component 1 scores were plotted against principal component 2 scores for each of the individuals.

### Association mapping

Marker-trait association was analyzed using linkage disequilibrium by the software TASSEL 4.0 (http://www.maizegenetics.net/). Both general linear model (GLM) and mixed linear model (MLM) were used to identify marker-trait association in the PIO and AFX populations [37]. For GLM analysis, only marker and trait data were used for analyzing. For MLM, the kinship (K) matrix generated by TASSEL was incorporated in the association analysis. TASSEL calculates kinship as the proportion of alleles shared between each pair of lines. Once this matrix is calculated, the numbers are rescaled so that the numbers fall between 0 and 2 (Peter Bradbury, Personal communication). A p-value of <0.05 was used as the threshold for calling the significant markers associated with VW resistance.

## Results

### Bulk segregant analysis

SSR markers that showed polymorphisms between the resistant and susceptible bulks were used for genotyping all individuals of each R and S bulks. They were scored as ‘1’ for presence and ‘0’ for absence. Frequencies that appeared to deviate from random distribution between bulks were evaluated using a Χ^2^ test and frequencies with a p-value <0.05 were considered significant. Chromosomes 1, 2, 4, 7, and 8 contained SSR marker alleles significantly associated with resistance to Verticillium wilt based on this criterion (Table 1). A total of 14 significant markers were identified, of those, two were located on chromosome 1. Marker MtBC01G06F3 were significantly associated with VW resistance in two bulks (CW20108 and CW065006) from the Alforex populations. Marker MtBB30E02 was significant in bulk V9 from Forage Genetics populations. Three markers were significant on chromosome 2 and they were identified in the V9 buck as well. Only one marker, AW261 was significant on chromosome 4, however, it was identified in two bucks V9 and V11 from Forage Genetics populations. Seven significant SSR markers were identified on chromosome 7, three of them (AW212, BF141 and BG288) were identified in two bulks. Marker AW255 located on chromosome 8 was significant in two bulks from the Alforex populations (Table 1).

### Phenotypic analysis of two biparental populations

**Figure 1:**
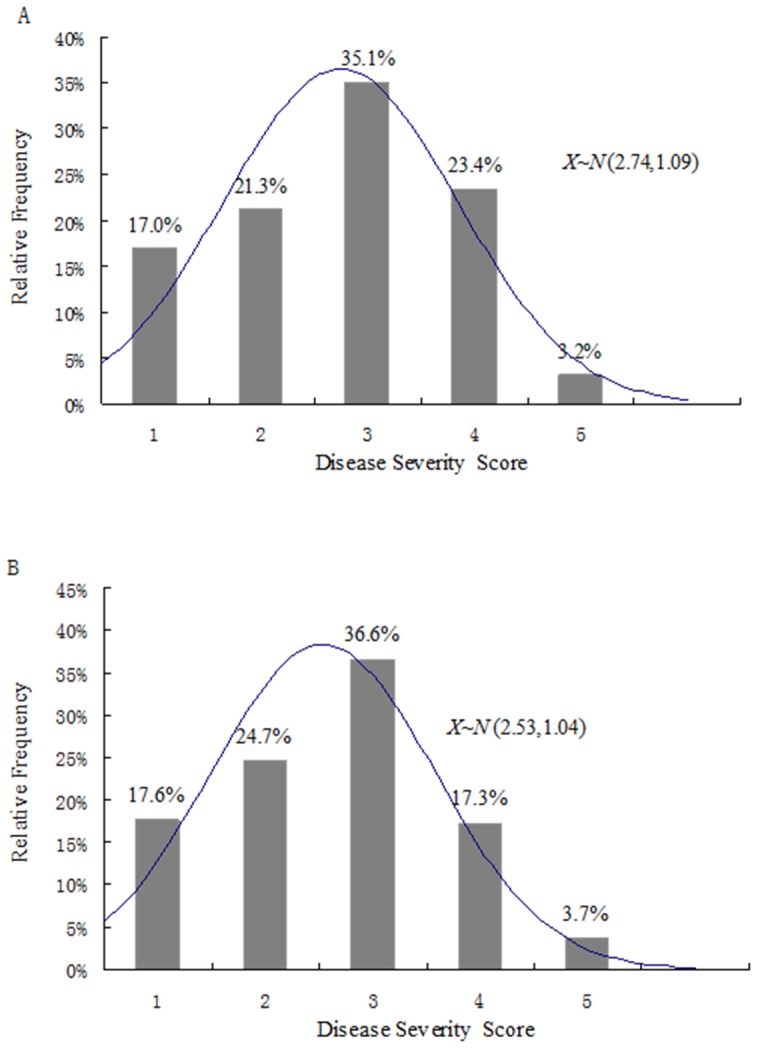
Distribution of disease severity scores in the Pioneer (A) and Alforex populations (B). 1 =  resistant with no or minimal chlorosis of lower leaves; 2 =  moderately resistant with chlorosis of lower and middle leaves but no chlorosis of terminal leaves; 3 =  susceptible with well developed symptoms of chlorosis, and necrotic and twisted terminal leaflets on at least one, but not all main stems, 4 =  susceptible with severe symptoms of chlorosis, necrosis, and twisting of all leaflets on all main stems; and 5 =  dead plant.

The foliar VW symptoms of parents and F1 progeny were evaluated after pathogen inoculation in the greenhouse. The means of Verticillium wilt disease severity scores were 2.74 and 2.54 for the PIO and AFX populations, respectively. The P-values for equality of variance (Levene's Test) and means (T-test) were greater than 0.05, suggesting no significant difference between the two populations (Table 2). Thus, they can be combined into one data set for association mapping. The frequency of disease classes in the PIO and AFX populations exhibited a normal distribution (Fig. 1 A and B). In addition, the p-values of the Kolmogorov-Smirnov test were more than threshold (0.05) (Table 3), confirming the hypothesis of a normal distribution of disease severity scores in the PIO, AFX and the combined populations.

**Table 2:** Comparison of means and variance of disease severity scores from two F_1_ mapping populations (PIO and AFX) inoculated with *Verticillium alfalfae*.

Population	No.*	Mean	Standard error	Levene's test for equality of variances	T-test for equality of means#
PIO	188	2.75	0.08	p = 0.513	p = 0.08
AFX	164	2.54	0.08		

Note: “*”, number of individuals in each population. “#”, means of disease severity scores.

**Table 3:** Evaluation of disease severity scores from two F_1_ mapping populations (PIO and AFX) for normal distribution using the Kolmogorov-Smirnov tests.

Population	Kolmogorov-Smirnov Z	p-value	Significance
PIO	0.443	0.989	ns
AFX	0.354	1.000	ns
Combined*	0.474	0.978	ns

Note: “*”, data of PIO and AFX populations were combined and analyzed. “ns”, not significant.

### SNP genotyping using HRM

**Figure 2:**
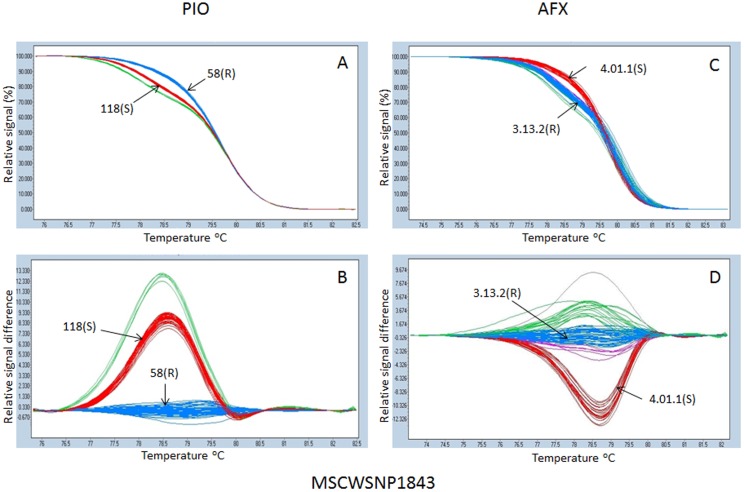
High resolution melting profiles of SNP markers MSCWSNP1843 in the Pioneer (PIO) (A, B) and Alforex (AFX) (C, D) populations. The real time PCR was performed by LightCycler 480) and the HRM profiles were analyzed using the gene scanning module software. Normalized melting curves (A, C) and temperature shift difference plots (B, D) are presented. Three groups of genotypes were distinguished by three colors (red, blue and green). Melting curves for parents in each population are indicated by arrows. Resistant parents: 58(R)  =  55V50-58(R) (Pioneer) and 3.13.2 (R)  =  CW065006-3-13-2 (R) (Alforex), susceptible parents: 118(S)  =  55V50-118(S) (Pioneer) and 4.01.1(S)  =  CW065006-4-01-1(S) (Alforex).

**Figure 3:**
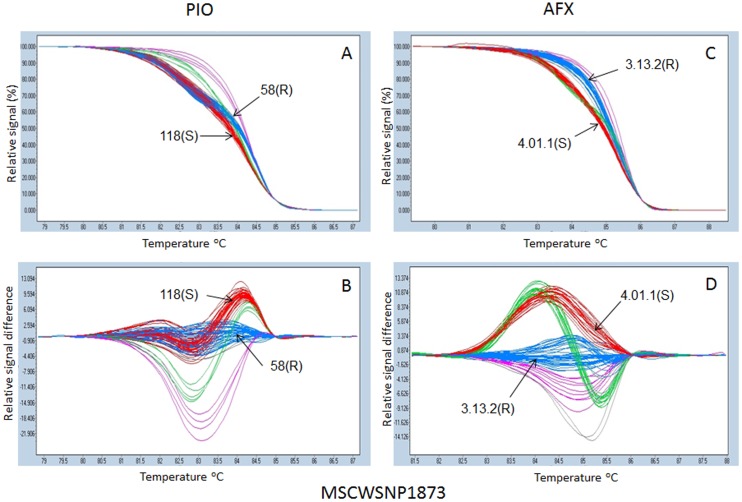
High resolution melting profiles of SNP markers MSCWSNP1873 in the Pioneer (PIO) (A, B) and Alforex (AFX) (C, D) populations. The real time PCR was performed by LightCycler 480) and the HRM profiles were analyzed using the gene scanning module software. Normalized melting curves (A, C) and temperature shift difference plots (B, D) are presented. Five colors (red, blue, green, pink and grey) represent five distinct groups of genotypes. Melting curves for parents in each population are indicated by arrows. Resistant parents: 58(R)  =  55V50-58(R) (Pioneer) and 3.13.2 (R)  =  CW065006-3-13-2 (R) (Alforex), susceptible parents: 118(S)  =  55V50-118(S) (Pioneer) and 4.01.1(S)  =  CW065006-4-01-1(S) (Alforex).

SNP genotyping using HRM enabled the identification of different clusters corresponding to different allelic classes of the autotetraploid alfalfa populations evaluated. Genotypic classes were visualized as normalized melting curves (Figs. 2 and 3, A and C) and normalized melting difference plots (Figs. 2 and 3, B and D). Genotypes with similar HRM profiles were clustered into the same group (Figs. 2 and 3) and HRM profiles at a given locus were used to assign individuals to genotyping groups. Figs. 2 and 3 shows examples of HRM profiles of two significant SNP markers, MSCWSNP1842 and MSCWSNP1873, respectively. The resistant and susceptible parents from each of the two populations were clustered into different groups at the given SNP markers. However, not all marker loci could be classified into 5 allelic groups similar to MSCWSNP1873 (Fig. 3 C and D), as was the case for MSCWSNP1843 (Fig. 2 A and B) with only three distinct groups.

### Population structure

**Figure 4:**
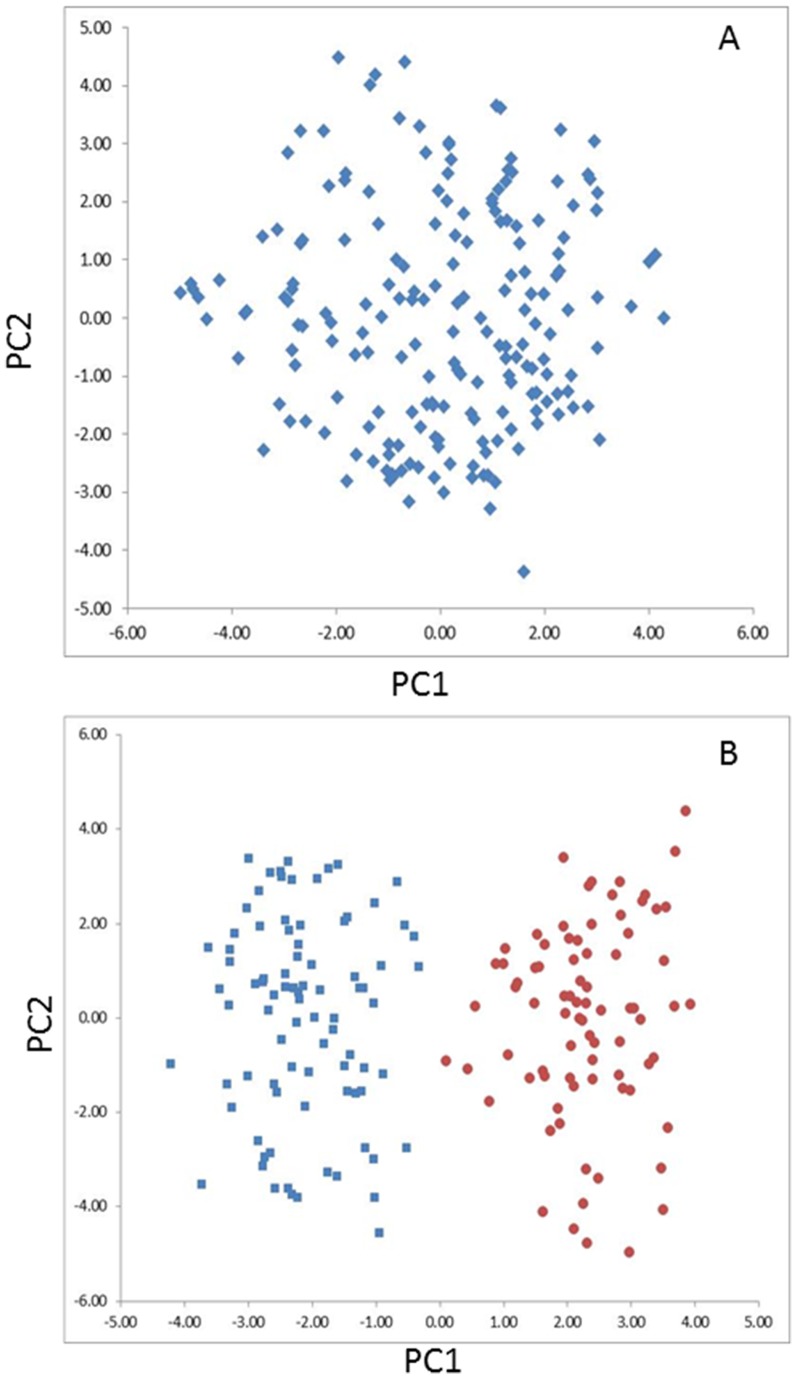
Principal components analysis of marker data in the Pioneer (A) and Alforex populations (B). The first and second principal components were used for graphing. Each data point represents a genotype. No subpopulation was detected in the Pioneer population (A), while two clusters were distinguishable in the Alforex population (B).

The result obtained from the principal component analysis suggests that no subpopulations exist in the PIO population (Fig. 4 A). However, two subpopulations represented as two clusters were found in the AFX population based on the PCA (Fig. 4 B). Population structure was therefore taken into account in the mixed linear model using a kinship matrix to avoid identifying spurious association that is not linked to any causative loci during the association mapping analysis.

### Identification of marker-trait association

Marker-trait association was analyzed in the PIO and AFX populations separately and in combination using both the GLM and MLM. Six significant markers (p<0.05) were identified in the PIO population (MSCWSNP0367, 0816, 1176, 1481, 1791, 1867, and 1873) (Table 4). Three of these markers (MSCWSNP0367, 0816 and 1791) were located on chromosome 7. Two markers (MSCWSNP1176 and 1867) located on chromosome 8 were identified. Marker MSCWSNP1867 showed the highest significant correlation coefficient with Verticillium wilt resistance (R^2^ = 0.12, p = 0.00025). Six significant markers associated with VW resistance were identified in the AFX population and they were located on chromosomes 2, 4, 7, and 8 (Table 4). MSCWSNP1481 located on chromosome 2 was the only marker identified in both the PIO and AFX populations. Marker MSCWSNP1821 identified from the AFX population were located on the same chromosomal region (Chr. 7) as did markers MSCWSNP0816 identified in the PIO population. They were likely to identify the same locus. On chromosome 8, Marker MSCWSNP1843 identified in the AFX was located in a different region compared to MsCWSNP1176 and 1867identified in the PIO population, suggesting that they may identify different loci (Table 4). To increase the population size, we combined the two populations and analyzed marker-trait association. Increased population size improved the power of detection and we identified 10 SNP markers significantly associated with Verticillium wilt resistance (p<0.001) in the combined population, with a total of R^2^ = 0.76 (Table 4). These markers were spread out on 5 chromosomes (Chr. 1, 2, 4, 7 and 8). A new marker, MSCWSNP0929 was identified on chromosome 1 in the combined population, whereas no significant marker was identified on chromosome 1 in the PIO or AFX population separately. Three markers (MSCWSNP 1481, 1843 and 2519) identified in the combined population were also identified in the PIO and AFX populations.

**Table 4:** Most significant SNP markers associated with Verticillium wilt resistance in the PIO, AFX and combined populations.

Population	Marker	Chr.	Position*	p-value	R^2^
PIO	MSCWSNP1481	2	14708250	4.41E-02	0.05
	MSCWSNP1791	7	4941313	2.52E-02	0.08
	MSCWSNP0367	7	20300221	1.85E-02	0.06
	MSCWSNP0816	7	29918333	3.97E-02	0.07
	MSCWSNP1867	8	13132620	2.51E-04	0.12
	MSCWSNP1176	8	19720655	1.46E-03	0.05
AFX	MSCWSNP1481	2	14708250	1.54E-02	0.07
	MSCWSNP0157	4	11518650	5.60E-03	0.10
	MSCWSNP2519	4	17321522	7.12E-03	0.11
	MSCWSNP1778	7	804111	5.29E-02	0.07
	MSCWSNP1821	7	27151713	1.24E-02	0.07
	MSCWSNP1843	8	585216	9.76E-03	0.07
Combined	MSCWSNP0929	1	1805680	1.37E-05	0.09
	MSCWSNP1481	2	14708250	1.58E-07	0.11
	MSCWSNP2519	4	17321522	3.35E-04	0.07
	MSCWSNP0265	7	736202	1.69E-04	0.08
	MSCWSNP1797	7	9591409	9.33E-04	0.07
	MSCWSNP1248	7	31361790	1.33E-05	0.08
	MSCWSNP1843	8	585216	5.76E-08	0.11
	MSCWSNP1854	8	5881455	8.86E-05	0.08
	MSCWSNP0386	8	24839922	4.76E-05	0.07

Note: “Chr.”, chromosome assigned based on the marker position in *Medicago truncatula*. “*”, the position of locus was based on *M. truncatula*. “Combined”, data of PIO and AFX populations were combined for association mapping.

**Figure 5:**
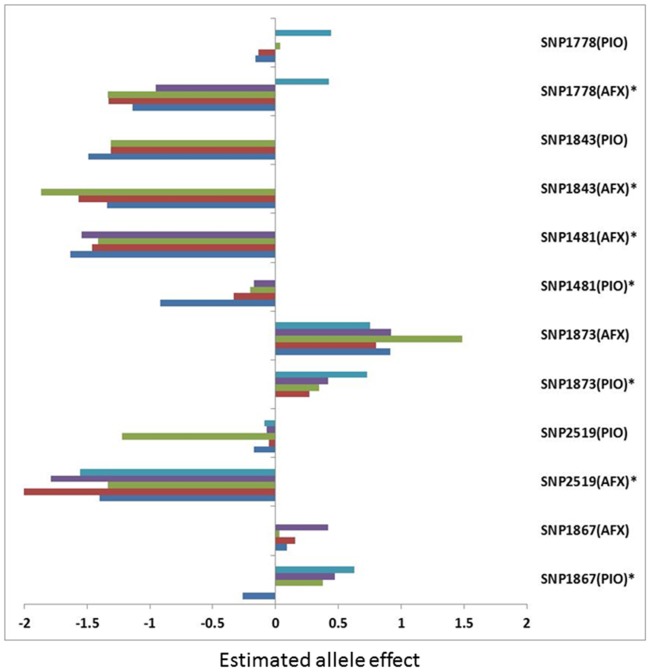
Comparison of allele effects of six significant SNP markers associated with Verticillium wilt resistance between the pioneer and Alforex populations. Estimated values were represented by bars and different allele types (up to 5 alleles per locus) were distinguished by different colors. Star “*” indicates the population in which the significant marker was identified.

To estimate marker effect in different resources, we estimated allele effects of most significant markers and compared them between the PIO and AFX populations. As shown in Fig. 5, positive and negative values of allele effects indicate sources of resistance. If the value is positive, the source of resistance at the locus is from R parent or vice versa. In general, similar patterns of allele effect were found between the PIO and AFX parents. For instance, at the SNP1873 locus, positive values were shown for all alleles in both PIO and AFX populations, indicating resources of resistance at this locus were from the R parents in both populations. In contrast, loci SNP1481, 1843 and 2519 had negative effects for all alleles and the resources of resistance for these loci were from the S parents in both populations. Marker SNP1778 had positive and negative values in both populations, suggesting the contributions of this locus to VW resistance were from both parents.

## Discussion

In this study, we first used bulk segregant analysis to identify chromosomes likely to contain markers associated with resistance to Verticillium wilt. The result of BSA provided information for choosing SNP markers in the target regions for genotyping by HRM in the second phase. We then used SNP genotyping focused on the targeted chromosomal regions to identified SNP markers for VW resistance in two alfalfa populations using association mapping. Given that the chemicals such as fluorescence dyes used in the HRM are expensive, the avoidance of using markers in non-target regions reduced cost. Our result indicates that the combination of BSA and HRM platforms for mapping disease resistance in alfalfa is feasible. Using this strategy, we identified 17 SNP markers associated with VW resistance in two alfalfa populations in relatively short time. The markers identified by HRM had similar chromosomal locations to those identified by BSA (Table1 and Table 4, chromosomes 1, 2, 4, 7 and 8), indicating a good agreement between the two analyses.

More significant markers were identified in the combined population (10 markers) than the separate PIO and AFX populations (6 markers each). Seven out of ten markers identified in the combined population were not identified in the separated populations. Among them, marker MSCWSNP0929 was the only one located on chromosome 1, whereas no significant marker was identified on chromosome 1 in the separated populations. This supports that increase the size of population improves the power of detection for marker-trait association. The association of locus MSCWSNP1481 to VW resistance was constantly identified in all populations, and a similar pattern of marker effects was found in the PIO and AFX populations, suggesting similar mechanism of VW resistance exits in two populations. On the other hand, a majority of markers identified in different chromosomal regions between the two populations may suggest alternate mechanisms of VW resistance between them.

Five significant markers identified on chromosome 8 explained 42% of the phenotypic variation, ant they are likely to represent novel VW resistance loci that have not been previously reported. However, these loci need to be validated. After validation, the markers linked to the resistance loci may be useful for developing diagnostic markers for marker-assisted selection for Verticillium wilt resistance in alfalfa.

Previous reports on the genetic basis of Verticillium wilt resistance in alfalfa suggested monogenic and quantitative resistance depending on the genetic background [38], [39]. Our results identified multiple loci associated with Verticillium wilt resistance, suggesting quantitative resistance in both mapping populations. Miller and Christie [40] reported that general combining ability (GCA) significantly influenced the expression of resistance to Verticillium wilt in ‘Vertus’ alfalfa and suggested that additive genetic variation is the primary source of Verticillium wilt resistance. This conclusion is supported by the results of the present study in which several putative QTL were identified for Verticillium wilt resistance. Papadopoulos [41] reported that variation in Verticillium wilt resistance was due primarily to additive gene effects in spite of some evidence of non-additive gene action.

QTLs associated with resistance to *Stagonospora meliloti* were identified in an autotetraploid alfalfa backcross population [11], and the largest locus contributed up to 17% of the phenotypic variation. Major QTLs related to resistance to *C. trifolii* have also been mapped in tetraploid alfalfa populations [13]. However, to date, no gene/QTL associated with Verticillium wilt resistance has been reported in tetraploid alfalfa. Given the level of sequence conservation between alfalfa and *M. truncatula*, one can envision the utility of the resistance genes or QTL identified in *M. truncatula* for alfalfa improvement [22], [42]. For example, QTLs for partial Verticillium wilt resistance were identified in two resistant parental lines including a major QTL on chromosome 7, and two minor QTLs on chromosomes 2 and 6 in *M. truncatula* [43]. In this study, three significant markers associated with Verticillium wilt resistance were identified on chromosome 7 and one on chromosome 2, suggesting similar locations for VW resistance in both species.

Although a good agreement of marker effects was found between different populations generated from two sets of parents (Fig. 5), further validation of marker effects is needed. We plan to test all significant markers identified in this study in a wide range of populations with known background of resistance to VW in alfalfa. Efforts aimed at development of high-throughput SNP genotyping platform such as genotyping by sequencing, for a comprehensive genome scan of segregating populations combined with additional phenotypic data for VW response will be used to validate the markers for VW resistance identified in this study as well as increase the likelihood of identifying additional loci contribute to VW resistance in alfalfa. Information on these loci and possible candidate genes underlying may provide insight into VW resistance gene network in *Medicago* species and accelerate the development of new alfalfa cultivars with resistance to Verticillium wilt.

## Supporting Information

S1 Table Simple sequence repeat (SSR) markers used for bulk segregant analysis.
(XLSX)

S2 Table Single nucleotide polymorphism (SNP) markers used for high resolution melting.
(XLSX)
